# Effects of dietary *Bacillus amyloliquefaciens* on the growth, immune responses, intestinal microbiota composition and disease resistance of yellow catfish, *Pelteobagrus fulvidraco*

**DOI:** 10.3389/fcimb.2022.1047351

**Published:** 2022-11-14

**Authors:** Mingyang Xue, Yeying Wu, Yizhan Hong, Yan Meng, Chen Xu, Nan Jiang, Yiqun Li, Wenzhi Liu, Yuding Fan, Yong Zhou

**Affiliations:** ^1^ Yangtze River Fisheries Research Institute, Chinese Academy of Fishery Sciences, Wuhan, China; ^2^ College of Life Sciences, Wuchang University of Technology, Wuhan, Hubei, China; ^3^ Department of Research and Development, Wuhan Dynamic Life Science Co, Ltd, Wuhan, Hubei, China; ^4^ Department of Production, Hainan Yonghe Biotechnology Co, Ltd, Qionghai, Hainan, China

**Keywords:** *Bacillus amyloliquefaciens*, intestinal microbiota, disease resistance, *Pelteobagrus fulvidraco*, yellow catfish, digestive enzyme activity, dietary supplementation

## Abstract

The influence of dietary probiotic *Bacillus amyloliquefaciens* on the growth performance, digestive enzyme activity, immune parameters and disease resistance of yellow catfish (*Pelteobagrus fulvidraco*) was evaluated. Commercial diet (C) or diet containing 10^6^ cfu/g *B. amyloliquefaciens* (T) was fed for 4 weeks, and final weight (FW), specific growth rate (SGR) and feed conversion ratio (FCR) were improved (*p<*0.05) in the T group. Dietary *B. amyloliquefaciens* increased protease and amylase activities in the digestive tract after 2 and 4 weeks, respectively. Respiratory burst (RB), plasma lysozyme (LZM) activity, total antioxidant capacity (T-AOC) and superoxide dismutase (SOD) activity were also elevated (*p<*0.05). Immune-related genes signal transducer and activator of transcription 1 (STATA-1), immunoglobulin M (IgM) and C-type lectin (CTL) were upregulated (*p<*0.05), but interleukin-1 β (IL-1β) was not (*p >*0.05). Intestinal microbiota analysis showed that the community structure was significantly different between the two groups; the relative abundance of *Cetobacterium* was increased but *Plesiomonas* was decreased in T. Moreover, challenge tests showed that the resistance of fish fed *B. amyloliquefaciens* against *Aeromonas veronii* and *Edwardsiella ictaluri* was significantly enhanced (*p<*0.05). In conclusion, dietary supplementation of *B. amyloliquefaciens* can effectively improve the growth performance, digestive enzyme activity, immune responses, intestinal microbiota composition and disease resistance of yellow catfish.

## 1 Introduction

Aquaculture is a rapidly growing industry, but rapid expansion of fish farming has resulted in the increased occurrence of diseases, and outbreaks have hindered aquaculture development (Bondad-Reantaso et al., 2005). There is increasing interest in the use of functional feeds that contain natural supplements such as probiotics and prebiotics to improve fish growth and health (Akhter et al., 2015; Huang et al., 2015; Dawood and Koshio, 2016). Diets supplemented with probiotics have been studied in many aquatic animals, such as tilapia (Rmm et al., 2020), shrimp (Sadat Hoseini Madani et al., 2018), grass carp (Liu et al., 2018; Qi et al., 2020) and crayfish (Xu et al., 2021), showing positive effects on improving water quality, increasing nutrient utilisation, enhancing immune status and disease resistance (Kiron, 2012; Yang et al., 2015; Hoseinifar et al., 2016). The most widely-investigated probiotics include *Bacillus* sp.*, Lactobacillus* sp. *and Saccharomyces cerevisiae* (Newaj-Fyzul et al., 2014; Akhter et al., 2015). *Bacillus* is a genus of Gram-positive, aerobic or facultative anaerobic, heat-stable spore-forming bacteria (Nakagawa et al., 2003; Hong et al., 2005). *Bacillus* sp. exhibit strong tolerance to environmental changes and antibacterial activities. These bacteria can also facilitate digestion, promote immune responses, and help maintain a balanced intestinal microbiota in hosts (Casula et al., 2002; Reda et al., 2017; Mingmongkolchai and Panbangred, 2018).

Yellow catfish (*Pelteobagrus fulvidraco*), a small teleost fish with exceptional flesh quality and high commercial value, is widely cultured in China. At present, yellow catfish ranks second among freshwater fish produced in 27 provinces of China (Wang et al., 2022), with yields reaching 5.8×10^5^ tons in 2021 (Fisheries and Fisheries Administration Bureau of Ministry of Agriculture and Industry, 2022). However, under intensive aquaculture conditions, bacterial infectious diseases occur frequently, resulting in severe economic losses (Ye et al., 2009). *Aeromonas veronii* and *Edwardsiella ictaluri* are two pathogenic bacteria in fish and could result in considerable economic losses in yellow catfish aquaculture (Zeng et al., 2021). *A. veronii* is a severe causative agent of ascites disease in yellow catfish (Zhou et al., 2019). *E. ictaluri* can cause “head perforation disease” in yellow-head catfish (Liu et al., 2010). Nevertheless, studies on using probiotics and prebiotics as dietary supplements for yellow catfish are lacking. Only Ming et al. (2016) reported studies of Taurine as a prebiotic in growth and immunity of yellow catfish (Ming et al., 2016).

The objective of the present study was to evaluate the probiotic properties of *B. amyloliquefaciens* on yellow catfish. Growth performance, digestive enzyme activities, immune responses, intestinal microbiota composition and disease resistance of yellow catfish were investigated. The results provide a solid base for future development of the yellow catfish farming industry.

## 2 Materials and methods

### 2.1 Fish and bacteria

Yellow catfish weighing 21 ± 1.2 g were obtained from a commercial yellow catfish farm located in Hubei province, China, and transported alive to Yangtze River Fisheries Research Institute, Chinese Academy of Fishery Sciences, Wuhan, China. Fish were adapted to the aquarium rearing conditions (400 L) for 14 days and fed a commercial fish feed (Tongwei, Chengdu, China). Water temperature (26 ± 0.5°C) was measured regularly during the experiment. Tanks were continuously aerated and 30% of water was renewed daily. All experimental procedures were conducted according to the guidelines of the Animal Experimental Ethical Inspection of Laboratory Animal Centre, Yangtze River Fisheries Research Institute, Chinese Academy of Fishery Sciences (ID Number: YFI2022-zhouyong-06).

**Figure 1:**
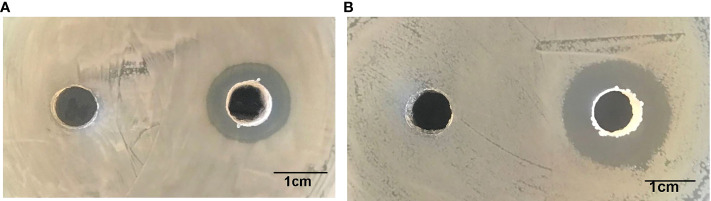
Inhibition zones of *B*. *amyloliquefaciens* against *E. ictaluri* **(A)** and *A*. *veronii* **(B)**.

The *B. amyloliquefaciens* strain was isolated from yellow catfish intestine and identified by cluster analysis of the 16S rDNA sequence (SRA: SRR21820145). The *B. amyloliquefaciens* strain was selected as a potential probiotic due to its antagonistic activity against the pathogenic strains *Aeromonas veronii* and *Edwardsiella ictalurid* (Qi et al., 2020), visualised as inhibition circles on Luria-Bertani (LB) plates (**Figure 1**). Prior to experiments, the bacterial strain was inoculated in LB broth medium and cultured at 30°C with shaking at 180 rpm for 24 h. Stock cultures were stored in LB broth medium containing sterile 30% (v/v) glycerol at -80°C.

### 2.2 Diet preparation

Commercial feed was used as basal diet. To prepare the supplementary diet, bacterial cells were harvested after overnight culture by centrifugation at 4°C for 15 min at 6000 g and washed three times with sterile phosphate-buffered saline (PBS, pH 7.4). Subsequently, cells were re-suspended in PBS at 10^6/^mL and mixed with basal diet. Control diet was prepared by adding the same volume of PBS to the basal diet. Diets were freeze-dried and stored at 4°C for further use (Gobi et al., 2018). Experimental diets were prepared every week to ensure the vitality of probiotics in diets.

### 2.3 Feeding trails

After an acclimatisation period, fish were randomly divided into control (C, fed basal diet) and treatment (T, fed *B. amyloliquefaciens* supplementation diet) groups. These groups were distributed in six tanks with 60 fish per tank. Each group included three replicates. Basal diet and *B. amyloliquefaciens-*enriched diet were given twice a day at 2% body weight for the experimental period (4 weeks).

### 2.4 Sample collection

During the 2^nd^ and 4^th^ week of feeding, nine fish from each tank were randomly collected, anaesthetised with MS222 (Sigma Aldrich, St. Louis, U.S.A) at 100 mg/L, blood was collected from the caudal vein of each fish with a 1 mL syringe, and placed in plastic Eppendorf tubes containing anticoagulant solution (heparin). The tubes were kept at 4°C overnight and centrifuged at 3000 g for 10 min and the obtained serum was stored at -80°C. During the 2^nd^ and 4^th^ week of feeding, intestine samples from three fish per group were collected to determine digestive enzyme activity, and midgut tissue from three fish per group was obtained to determine the relative mRNA levels of immune-related genes. During the 4^th^ week of feeding, intestine tissue was flash-frozen in liquid nitrogen and stored at -80°C for Illumina sequencing.

### 2.5 Growth measurements

Weight was measured at the beginning and end of the experiment. The growth performance of fish was calculated as follows:


$$Weight\;gain\;\left(g/fish\right)\;=\;W_{t}-\;W_{0}$$

$$Specific\;growth\;rate\;\left(SGR\right)\;=\;100\;\times \;\left(lnW_{t}-\;lnW_{0}\right)/t$$

$$Feed\;conversion\;ratio\;\left(FCR\right)\;=\;FI/\left(W_{t}-W_{0}\right)$$

where W_t_ and W_0_ are the final and initial weight, respectively, t is the duration of feeding (in days), and FI is feed intake.

### 2.6 Digestive enzyme analysis

Intestine samples were homogenised in PBS at a ratio of 1:9 (w/v) using a glass homogeniser at 4°C. The homogenate was centrifuged at 5000 × g for 20 min at 4°C to remove tissue debris (Safari et al., 2014). The supernatant was kept on ice and used within 24 h. Aliquots of the supernatant, designated as the crude extract, were used to estimate protease and amylase activities.

Total protein content was determined using bovine serum albumin as the standard according to the Bradford method (Bradford, 1976). Protease, amylase and lipase activities were assessed with a rapid colorimetric kit described by the manufacturer’s instructions (Jiancheng, Nanjing, China).

### 2.7 Immunological assays

#### 2.7.1 Respiratory burst activity

Generation of intracellular superoxide radicals by macrophages was determined from the reduction of nitro-blue tetrazolium (Solarbio, Beijing, China) as described previously (Hong et al., 2006), with absorption measured at 620 nm using KOH/dimethylsulphoxide (DMSO) as the blank.

#### 2.7.2 Serum immune responses

Plasma lysozyme (LZM) activity, total antioxidant capacity (T-AOC) and superoxide dismutase (SOD) activity were measured using appropriate kits according to the manufacturer’s instructions (Jiancheng, Nanjing, China).

#### 2.7.3 Changes in immune-related gene expression

Total RNA was extracted from tissues using TRIzol reagent (Invitrogen, Carlsbad, USA). The quality and purity of RNA were assessed by spectrophotometry, and 260:280 ratios were 1.8−2.0. Total RNA was reverse-transcribed into cDNA using a RevertAid First Strand cDNA Synthesis Kit (TaKaRa, Dalian, China) following the manufacturer’s instructions. Real-time quantitative PCR was performed using a SYBR Premix Ex Taq Perfect real-time Kit (TaKaRa) and a CFX96 Real-Time PCR Detection System (Bio-Rad, Berkeley, USA). The β-actin housekeeping gene served as an internal reference. Specific primers are listed in **Table 1**. In all cases, each PCR was performed with triplicate samples. The relative quantification of gene expression among groups was analysed by the 2^-ΔΔCT^ method (Livak and Schmittgen, 2001).

**Table 1:** Primers used to quantify relative gene expression.

Gene	Primer sequence	Product size	References
β-actin	F: 5’-CATCACCATCGGCAACGAGAGG-3’	119 bp	(Zeng et al., 2021)
	R: 5’-CGTCGCACTTCATGATGCTCTTG-3’		
IL1-β	F: 5’-CAGGACCTCTTCACTATCTT-3’	198 bp	(Zhu et al., 2017)
	R: 5’-TTCATTTCCACCTTTCAG-3’		
STAT-1	F: 5’-AAGCGAGGACTGAACACC-3’	254 bp	(Zhu et al., 2017)
	R: 5’-TTATCACTGAGCAGAGCCTTA-3’		
IgM	F: 5’-AGAGCCAAAGTGAGCATTA-3’	216 bp	(Zeng et al., 2021)
	R: 5’-CTTGGCAGGTGTATGTGG-3’		
CTL	F: 5’-TACAACGGCGACAAACTGGA-3’	134 bp	(Zeng et al., 2021)
	R: 5’-TCCGTGGGGTCAAAACTACG-3’		

### 2.8 Genomic DNA extraction and Illumina miSeq sequencing

Bacterial genomic DNA was extracted using a Bacterial DNA Kit (Omega, Norcross, USA) following the manufacturer’s instructions. The V3−V4 region of the bacterial 16S rRNA gene was amplified by PCR using specific primers 338F (5′-ACTCCTACGGGAGGCAGCA-3′) and 806R (5′-GGACTACHVGGGTWTCTAAT-3′) with barcodes in 50 μL reactions. Thermal cycling consisted of initial denaturation at 95°C for 1 min, followed by 30 cycles at 95°C for 30 s, 55°C for 30 s, and 72°C for 45 s, and a final extension at 72°C for 10 min. After separating by agarose gel electrophoresis, samples were assessed on an Illumina MiSeq PE250 high-throughput sequencing platform. All sequence reads were quality-filtered and assembled using the Mothur software package (Schloss et al., 2009). Reads were clustered into operational taxonomic units (OTUs) at 97% identity by RDP Classifier algorithm (http://rdp.cme.msu.edu/) (Wang et al., 2007). The abundance of corresponding OTUs in each region was calculated at the genus levels. Abundance-based coverage estimator (ACE), Chao, Shannon and Simpson alpha-diversity indices were analysed by Mothur (version v.1.30) (Elizabeth et al., 2009). Rank - abundance curve was performed with R statistical software (http://www.r-project.org/) with the aid of the packages Fields and Vegan (Bates et al., 2013).

### 2.9 Challenge tests

Strains *A. veronii* and *E. ictaluri* pathogenic to yellow catfish were used in challenge tests. After the feeding trial, fish in each diet group were assigned to six tanks, fish in three tanks for each group were intraperitoneally injected with 0.2 mL *A. veronii* (10^7^ cfu/mL), and fish in other tanks were intraperitoneally injected with 0.2 mL *E. ictaluri* (10^7^ cfu/mL). Infected fish were observed daily and mortality was recorded for 10 days. All dead fish were examined bacteriologically to determine the presence of the pathogen.

### 2.10 Statistical analysis

Data were analysed by one-way analysis of variance (ANOVA) and expressed as the arithmetic mean ± standard deviation (SD). Survival curves were estimated by the Mann-Whitney U test and Kaplan-Meier method (Bland and Altman, 1998). Differences were determined by Tukey’s test in SPSS statistical software (SPSS Inc., USA) with *p*-values<0.05 indicating significance.

## 3 Results

### 3.1 Growth parameters

Weight gain and specific growth rate of fish fed diet supplemented with *B. amyloliquefaciens* were significantly higher (*p<*0.05) than those fed control diet (**Table 2**). FCR was significantly higher in fish fed control diet (*p<*0.05; **Table 2**).

**Table 2:** Growth parameters of yellow catfish after 4 weeks feeding with control diet (Control, C) and diet supplemented with *B. amyloliquefaciens* (Treatment, T).

Growth parameters	Experimental diets	
	Control	Treatment
Initial weight (g)	21.2 ± 0.16^a^	21.2 ± 0.24^a^
Final weight (g)	25.1 ± 0.21^a^	26.8 ± 0.17^b^
SGR	0.60 ± 0.2^a^	0.84 ± 0.2^b^
FCR	1.15 ± 0.3^a^	0.98 ± 0.2^b^

Results are means ± standard deviation (SD). Values in each row with different superscripts indicate significant differences among treatments at p<0.05. SGR, specific growth rate. FCR, feed conversion ratio.

### 3.2 Digestive enzyme analyses

**Figure 2:**
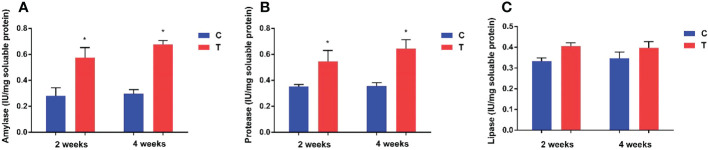
Effects of dietary *B*. *amyloliquefaciens* on amylase, protease and lipase activities in the gut of yellow catfish. Amylase **(A)**, Protease **(B)**, Lipase **(C)**. C, control group; T, treatment group. Results are means ± SD from three individual fish (*p<0.05).

Amylase and protease activities in the intestine of yellow catfish fed with *B. amyloliquefaciens* were increased significantly (*p<*0.05) compared with fish fed control diet (**Figure 2**). Amylase and lipase activities showed no significant differences between control and treatment groups (*p >*0.05).

### 3.3 Immunological assays

#### 3.3.1 Respiratory burst activity

**Figure 3:**
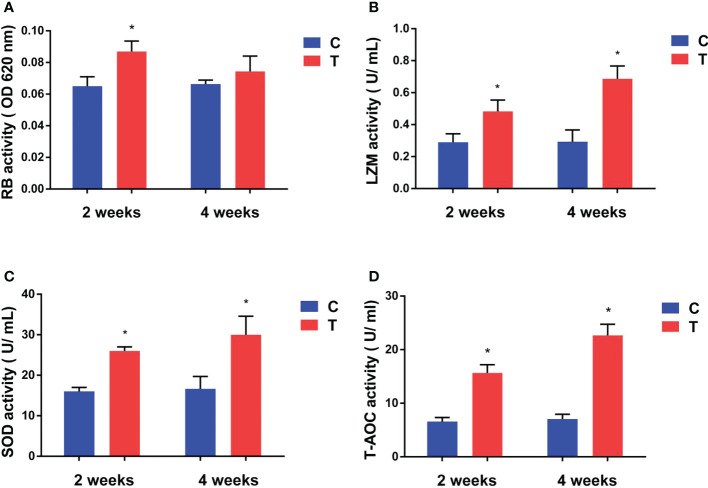
Effects of *B*. *amyloliquefaciens* on the respiratory burst (RB) activity **(A)**, lysozyme (LZM) activity **(B)**, superoxide dismutase (SOD) activity **(C, D)** total antioxidant capacity (T-AOC) of yellow catfish. C, control group; T, treatment group. Results are means ± SD from three individual fish (*p<0.05).

After yellow catfish were fed diet containing *B. amyloliquefaciens*, their respiratory burst (RB) activity increased significantly after 2 weeks (*p<*0.05) compared with the control diet group, but no statistically significant difference was found after 4 weeks (*p >*0.05; **Figure 3A**).

#### 3.3.2 Serum immune response analysis

LZM, and SOD activities and T-AOC of yellow catfish were increased significantly in the treatment diet group compared with the control group after 2 and 4 weeks of feeding (*p<*0.05; **Figures 3B–D**).

#### 3.3.3 Immune-related gene expression

**Figure 4:**
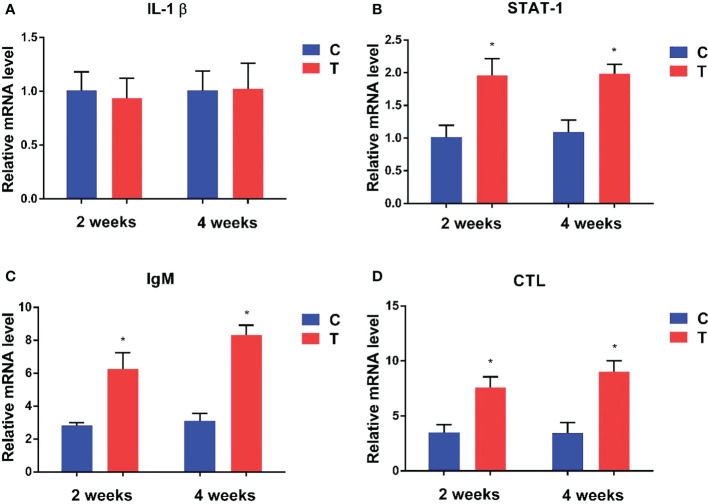
Relative expression levels of immune-related genes in the intestine of fish treated with *B*. *amyloliquefaciens* and *P. fulvidraco.* IL-1β **(A)**, STAT-1 **(B)**, IgM **(C)**, CTL **(D)**. C, control group; T, treatment group (*p<0.05).

Expression levels of immune-related genes at the 2^nd^ and 4^th^ week in the intestine of yellow catfish supplemented with different levels of *B. amyloliquefaciens* are presented in **Figure 4**. Signal transducer and activator of transcription 1 (STAT-1), immunoglobulin M (IgM) and C-type lectin (CTL) were significantly upregulated in fish fed with *B. amyloliquefaciens* compared with control diet (*p<*0.05). By contrast, interleukin-1β (IL-1β) mRNA levels were not significantly different among control and treatment groups (*p >*0.05).

### 3.4 Intestinal microbiota composition

**Figure 5:**
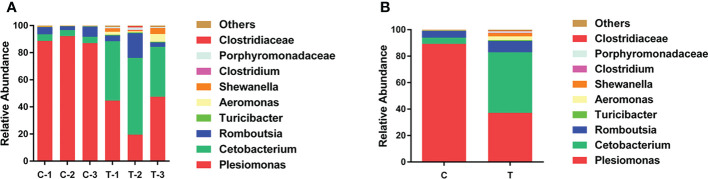
Structure and composition of the intestinal bacterial communities in yellow catfish at the genus level. **(A)** appearing in each sample, **(B)** means representing as two groups. C and C1−C3, control groups; T and T1−T3, treatment groups.

To explore changes in the gut microbiota community of yellow catfish fed a diet supplemented with *B. amyloliquefaciens*, OTUs of the intestinal microbiota were determined for each group according to the abundances of taxa at the genus level. At the genus level, the primary intestinal microbiota in all groups were *Plesiomonas*, *Cetobacterium* and *Romboutsia* (**Figure 5**). The relative abundance of *Cetobacterium* was increased significantly in the gut of yellow catfish fed *B. amyloliquefaciens* (*p<*0.05). Conversely, *Plesiomonas* was decreased significantly in the gut of yellow catfish fed *B. amyloliquefaciens* (*p<*0.05; **Figure 5**).

### 3.5 Richness and diversity of the intestinal microbiota

**Figure 6:**
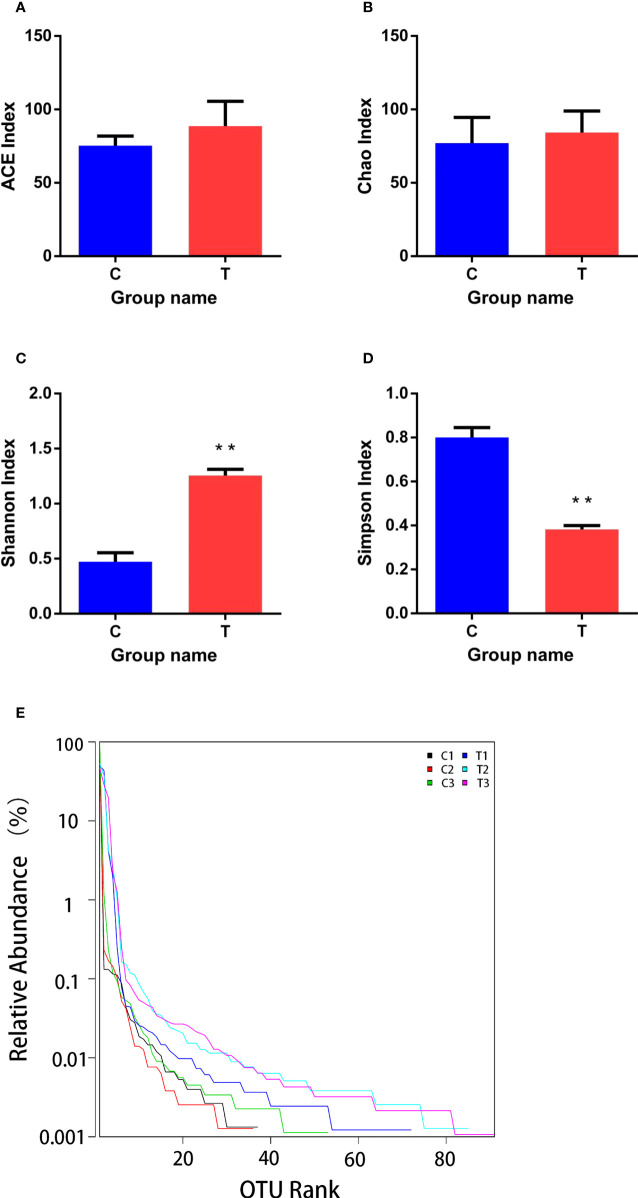
Richness and diversity of bacterial species in treatment and control groups. **(A–D)** are ACE, Chao, Shannon and Simpson indices of OTUs, respectively. **(E)** was Rank - abundance curve. C, control group; T, treatment group. Results are means ± SD from three individual fish (**p<0.01).

Good’s coverage ranged from 0.996 to 0.999, which indicated that the gut microbiota of the samples was reliably identified. There were no significant differences between ACE and Chao1 indices among control and treatment groups (*p >*0.05; **Figures 6A, B**). However, Shannon and Simpson indices were significantly higher and lower (*p<*0.01), respectively, in the treatment group than the control group (**Figures 6C, D**). The range of the curve on the horizontal axis in the treatment group was significantly higher than that in the control group (*p<*0.05; **Figure 6E**).

### 3.6 Challenge test

**Figure 7:**
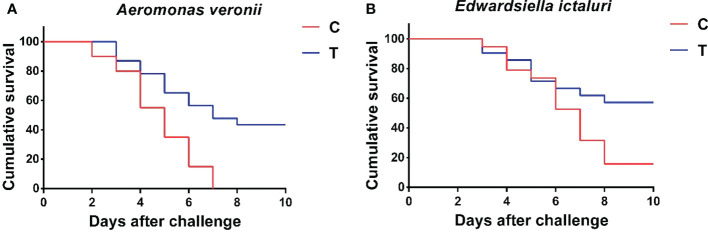
Cumulative survival rate of *P. fulvidraco* after challenge with *A. veronii* **(A)** and *E. ictaluri* **(B)** for 10 days. C, control group; T, treatment group.

The cumulative survival rates of yellow catfish challenged with *A. veronii* and *E. ictaluri* for 10 days are shown in **Figure 7**. At the end of the 10-day challenge test, the cumulative survival rate of fish fed with *B. amyloliquefaciens* diet was significantly higher than that of fish fed with control diet (*p<*0.05). Furthermore, *A. veronii* and *E. ictaluri* were respectively re-isolated from the artificially infected fish.

## 4 Discussion

Research on the dietary supplementation of probiotics in aquaculture is receiving increasing attention due to the demand for eco-friendly prophylactic measures for fish growth performance and health improvement (Geng et al., 2011; Lee et al., 2016; Asaduzzaman et al., 2018). Probiotics, such as *Bacillus* spp., are being used increasingly in aquaculture (Gobi et al., 2018). The effects of dietary supplementation of *Bacillus subtilis* HAINUP40 on growth, immunity and disease resistance of tilapia (*Oreochromis niloticus*) were investigated (Liu et al., 2017). In another study, Muthukrishnan et al. (2016)found that *Bacillus cereus* BP-MBRG/1b significantly improved growth performance, intestinal propionic acid production and haemolymph SOD activity in prawns (Muthukrishnan et al., 2021). In the present study, dietary supplementation of *B. amyloliquefaciens* improved growth performance, immune responses, the structure of the intestinal microbiota and resistance to pathogens in yellow catfish.

Previous studies show that many *Bacillus* spp. can effectively improve the host’s growth performance, such as percentage weight gain and feed efficiency. For instance, Madani et al. reported that oral administration of commercial probiotic *Bacillus* (*B. subtilis* and *B. licheniformis*) had beneficial effects on growth performance parameters and feed utilisation in *Litopenaeus vannamei* post-larvae (Sadat Hoseini Madani et al., 2018). Similarly, *B. amyloliquefaciens* significantly improved the specific growth rate and percentage weight gain of yellow catfish in the present study. It is known that increasing body weight gain in fish fed a probiotic supplemented diet can be attributed to increased digestive enzyme activity (Irianto and Austin, 2010). In the present study, *B. amyloliquefaciens* dietary supplementation significantly increased amylase and protease activities of yellow catfish. However, the proportion of enzymes produced by probiotics cannot be assessed, since probiotics can produce enzymes and may also stimulate the production of endogenous enzymes in fish (Wu et al., 2012; Dawood et al., 2016; Liu et al., 2017). Our results revealed that *B. amyloliquefaciens* could enhance the digestive enzyme activity of yellow catfish, and thus improve growth.

RBs produced by phagocytes are considered an important indicator of cellular immunity mechanisms in fish when evaluating their defence abilities against pathogens (Wu et al., 2015). Our study revealed that RBs of phagocytes in the experimental group were increased significantly after 2 weeks of feeding on a *B. amyloliquefaciens-*supplemented diet. LZM is an important component of the innate immune defence system against invasive pathogens. The rise of serum LZM levels suggests the elevation of various humoral factors that can protect the host during pathogen invasion. Liu et al. (2017) demonstrated that LZM activity of tilapia was increased significantly after fish were fed with *B. subtilis* HAINUP40 for 8 weeks (Liu et al., 2017). A similar result was observed in our study; feeding on *B. amyloliquefaciens* significantly increased LZM activity in yellow catfish. Therefore, *B. amyloliquefaciens* may induce disease resistance in yellow catfish against *A. veronii* and *E. ictaluri* by promoting RB and LZM activities.

Under normal physiological conditions, animal cells maintain a balance between generation and removal of reactive oxygen species. T-AOC includes enzymatic and non-enzymatic antioxidant activities. SOD is the first line of the antioxidant enzymatic defence system, and T-AOC is useful to evaluate the capacity of all antioxidants. In the present study, activities of SOD and TAOC were increased in the serum of yellow catfish whose diets contained *B. amyloliquefaciens*. SOD and T-AOC are indicators of the antioxidant status of fish, and are utilised as oxidative stress biomarkers. Therefore, oral administration of *B. amyloliquefaciens* promoted the antioxidant activity of yellow catfish. Similarly, Esteban et al. (2014) demonstrated that oral administration of *Shewanella putrefaciens* and *Bacillus* on gilthead significantly enhanced its SOD activity (Esteban et al., 2014). Significantly higher SOD levels were reported after 8 weeks of feeding on *Lactobacillus rhamnosus* in red sea bream (Dawood et al., 2016) and *B. subtilis* HAINUP40 in tilapia (Liu et al., 2017).

Cytokines are cell signalling molecules involved in many physiological processes, including the regulation of immune and inflammatory responses, which are important to maintain the health of hosts (Xiao et al., 2018). In the present study, mRNA expression levels of immune-related genes in intestine were measured after dietary administration of probiotic *B. amyloliquefaciens*. Pro-inflammatory cytokines such as IL-1β mediate powerful inflammatory responses in fish after infection (Julio et al., 2014). Our results showed that *Bacillus* did not cause inflammation in yellow catfish. Additionally, oral administration of *B. amyloliquefaciens* upregulated the expression of IgM, CTL and STAT-1 in intestine of yellow catfish. IgM and CTL play key roles in controlling pathogens and maintaining homeostasis in fish. A number of probiotics can effectively modulate the expression of inflammatory cytokines in many animals (Rebeca et al., 2013; Guo et al., 2016). These results suggest that immune cytokines are influenced by *B. amyloliquefaciens*, and further promote disease resistance in yellow catfish.

The intestinal microbiota plays important roles in host health due to its critical influence on metabolism and immune function (Xu et al., 2022). The richness and diversity of gut bacteria are closely linked to the stability of intestinal microbial communities in animals (Piazzon et al., 2019; Xue et al., 2022). Significantly increased Shannon index and significantly decreased Simpson index values were observed for the treatment group in the present study, while ACE and Chao1 index values among control and treatment groups were not significantly different. These results indicate that the diversity of the intestinal microbiota in treatment group was significantly increased, but there was no significant change in bacterial richness. *B. amyloliquefaciens* may improve intestinal stability and health by increasing the diversity of intestinal microbiota. As the largest immune organ in the body, the intestinal tract plays an important role in reducing the invasion of pathogenic bacteria. An increase in the diversity and stability of gut microbes also contributes to host immunity, this also contributes to the host’s resistance to disease. The relative abundance of *Cetobacterium* was increased significantly and *Plesiomonas* was decreased significantly in the gut of treated yellow catfish vs. controls. *Cetobacterium* is an important beneficial bacteria in the gut of aquatic animals, and it can produce large quantities of vitamin B-12 (Tsuchiya et al., 2008). Members of the genus *Plesiomonas* are ubiquitous opportunistic pathogens in aquaculture systems, and can cause infections in humans (Ekundayo and Okoh, 2019; Xi et al., 2019). In the present study, diets containing *B. amyloliquefaciens* increased the proportion of beneficial bacteria and decreased the proportion of harmful bacteria in yellow catfish.

The current study revealed a higher survival rate in *P. fulvidraco* challenged with *A. veronii* or *E. ictaluri* when fed a *B. amyloliquefaciens*-supplemented diet compared with a basal diet. The ability of probiotics to inhibit the growth of pathogenic bacteria and elevate the immune response of hosts might be important for reducing the percentage cumulative mortality and protecting yellow catfish against these pathogens.

## 5 Conclusions

In summary, *B. amyloliquefaciens* exhibits many properties of a good probiotic, including the ability to secrete extracellular enzymes and inhibit the growth of pathogenic bacteria. Oral administration of *B. amyloliquefaciens* can improve growth performance, digestive enzyme activities, immune responses, the structure of the intestinal microbiota and disease resistance against *A. veronii* and *E. ictaluri* in yellow catfish. These results indicate that *B. amyloliquefaciens* can be used as a potential probiotic in yellow catfish farming.

## Data availability statement

The data presented in the study are deposited in the NCBI (https://www.ncbi.nlm.nih.gov/) repository, accession number SRR21820145.

## Ethics statement

The animal study was reviewed and approved by Animal Experimental Ethical Inspection of Laboratory Animal Centre, Yangtze River Fisheries Research Institute, Chinese Academy of Fishery Sciences (ID Number: YFI2022-zhouyong-06).

## Author contributions

MX conceived and designed the study, performed the data collection, analysis, statistical analysis, and wrote the manuscript. YW and CX conducted the software analysis and literature review. YH and NJ conducted the animal management and sample collections. YM, WL and YL performed the microbial analysis, immunity analysis, and literature review. YF and YZ contributed to acquisition of funding, conceptualization, writing - review & editing, and supervision. All authors contributed to the article and approved the submitted version.

## Funding

This work was supported by the National Key Research Development Program of China (2019YFD0900105), and the Central Public-interest Scientific Institution Basal Research Fund (2020TD44).

## Acknowledgments

We thank Wuhan Dynamic Life Science and the Hainan Yonghe Biotechnology Co., Ltd. for support in carrying out this study.

## Conflict of interest 

Author YW is employed by Wuhan Dynamic Life Science Co, Ltd. Author YH is employed by Hainan Yonghe Biotechnology Co, Ltd.

The remaining authors declare that the research was conducted in the absence of any commercial or financial relationships that could be construed as a potential conflict of interest.

## Publisher’s note

All claims expressed in this article are solely those of the authors and do not necessarily represent those of their affiliated organizations, or those of the publisher, the editors and the reviewers. Any product that may be evaluated in this article, or claim that may be made by its manufacturer, is not guaranteed or endorsed by the publisher.
